# 

**Published:** 1877-04

**Authors:** 


### EDITORIAL, ETC.

---

*Southern Dental Association.*—The Ninth Annual Meeting of this Association will be held at Montgomery, Alabama, commencing Tuesday, April 10th 1877, and continue three or four days. A full attendance of the members is requested, as important business pertaining to the Association will be considered and acted upon. A cordial invitation is extended to all in the profession to attend this meeting.

Subjects for Discussion.—Dental Education, Relations of Dentistry to Medicine, Dentistry as a Profession, Causes of the Deterioration of the Teeth, Care of the Teeth during Gestation and Lactation, Deciduous Teeth—their Relation to the Permanent, and their Treatment, Transplanting and Replanting Teeth, Relative Merits of Cohesive and Non-cohesive gold as a Filling for Teeth, Treatment of Exposed Nerves, Dead Teeth, and Alveolar Abscess, Relative Merits of Rubber and Celluloid, The Arrangement of Dental Offices in regard to Health and Convenience.

Officers:

*President*, W. G. Redman, Louisville, Ky.
1*st Vice President*, G. C. Sandusky, Shelbyville, Tenn.
2*nd Vice President*, E. S. Chisholm, Tuscaloosa, Ala.
3*rd Vice President*, R. Russell, Nashville, Tenn.
*Corresponding Secretary*, W. L. Dismuskes, Nashville, Tenn.
*Recording Secretary*, H. E. Beach, Clarksville, Tenn.
*Treasurer*. S. J. Cobb, Nashville, Tenn.
*Executive Committee*: Samuel Rambo, L. C. Chisholm, and A. C. Ford.

appliances, increases also the probability of still other improvements. The inventor's royalty steps in to encourage the good work, and to secure the preservation of valuable suggestions and devices, now commonly lost to the profession. The benefit that would ultimately accrue to the profession from and through this line of advancement, is quite incalculable."

---

*A New Medical Journal.*—We have received the Prospectus of a New Medical Publication, to appear on the first day of May, 1877, under the title of "Maryland Medical Journal," and to be conducted by Drs. H. E. T. Manning and T. A. Ashby. It is to be issued monthly, and devoted to the advancement of Medicine in all its branches. Each number will contain not less than forty pages, and the subscription price is to be $3.00 per annum, free of postage. We wish the new enterprise every success.

---

### MONTHLY SUMMARY.

### TO READERS AND CORRESPONDENTS.

Communications intended for publication, and exchange journals and papers, should be addressed to the EDITOR OF THE AMERICAN JOURNAL OF DENTAL SCIENCE, 259 N. Eutaw St., Baltimore, Md. All letters relating to the business of the journal, or containing cash remittances, to be directed to Messrs. SNOWDEN & COWMAN. Articles for publication should be received by the editor at least three weeks previous to the first day of the month on which the number in which it is designed for them to appear, is issued.

☞The American Journal of Dental Science will contain fifty pages of reading matter in each number, making a volume of about

*Six Hundred* pages

EXCLUSIVE OF ADVERTISEMENTS

THE

### AMERICAN JOURNAL

OF

### DENTAL SCIENCE.

F. J. S. GORGAS, M. D., D. D. S., Editor.

The *Journal* is issued on the first of each month and contains not less than *fifty* pages of reading matter in each number, exclusive of advertisements, making a volume of *six hundred* pages per annum.

In each number will be found Original Communications, Correspondence, Selected Articles, Monthly Summary, Bibliographical Notices and Editorial Matter, and every effort will be exerted to render the *Journal* worthy of the patronage of the Dental profession.

A generous co-operation is respectfully solicited from the members of the profession; and as the *Journal* has now a printing office of its own, which materially lessens the cost of its publication, it will be issued at the reduced subscription price of

**$2.50 Per Annum in Advance.**

Communications are respectfully solicited on all subjects pertaining to the practice of dentistry, as well as reports of cases occurring in dental practice.

### RATES OF ADVERTISING.

| | 1 MO. | 2 MO. | 3 MO. | 6 MO. | 12 MO. |
|---|---|---|---|---|---|
| 1 PAGE. | $15 00 | $22 50 | $30 00 | $50 00 | $100 00 |
| ½ " | 10 00 | 15 00 | 20 00 | 30 00 | 50 00 |
| ¼ " | 7 00 | 11 00 | 15 00 | 20 00 | 30 00 |
| ⅛ " | 5 00 | 8 00 | 11 00 | 15 00 | 20 00 |

All original communications designed for publication, works for review, new instruments for notice, exchanges, &c., should be directed to the editor, 259 N. Eutaw St., Baltimore, Md.

All advertisements and subscriptions should be addressed to

SNOWDEN & COWMAN,

*Publishers of the Am. Journal of Dental Science.*

86 W. FAYETTE ST. BET. CHARLES & LIBERTY STS.,

BALTIMORE, MD.

